# Knowledge of Emergency Management of Avulsed Teeth among Italian Dentists-Questionnaire Study and Next Future Perspectives

**DOI:** 10.3390/ijerph18020706

**Published:** 2021-01-15

**Authors:** Marta Mazur, Maciej Jedliński, Joanna Janiszewska-Olszowska, Artnora Ndokaj, Roman Ardan, Gianna Maria Nardi, Roberto Marasca, Livia Ottolenghi, Antonella Polimeni, Iole Vozza

**Affiliations:** 1 Department of Oral and Maxillofacial Surgery, Sapienza University of Rome, Via Caserta 6, 00166 Rome, Italy; artnora.ndokaj@uniroma1.it (A.N.); Giannamaria.Nardi@Uniroma1.It (G.M.N.); livia.ottolenghi@uniroma1.it (L.O.); antonella.polimeni@uniroma1.it (A.P.); iole.vozza@uniroma1.it (I.V.); 2 Department of Interdisciplinary Dentistry, Pomeranian Medical University in Szczecin, al. Powstańców Wielkopolskich 72, 70-111 Szczecin, Poland; Maciej.jedlinski@pum.edu.pl (M.J.); jjo@pum.edu.pl (J.J.-O.); 3 Department of Economic Sciences, Koszalin University of Technology, Kwiatkowskiego 6e, 75-343 Koszalin, Poland; roman.ardan@tu.koszalin.pl; 4 Pediatric Dentistry Unit, Head and Neck Integrated Department, AOU Policlinico Umberto I of Rome, Viale Regina Elena 287/b, 00161 Rome, Italy; R.Marasca@policlinicoumberto1.it

**Keywords:** trauma management, avulsion of permanent teeth, Italian dentist, questionnaire, knowledge, international guidelines, IADT, dental avulsion, survey

## Abstract

(1) Background: In Italy, about one fourth of all schoolchildren experience a trauma to the permanent dentition. Management of avulsion trauma is challenging and requires adherence to clinical protocols. The aim of this study was to investigate the management knowledge of avulsed teeth among Italian dentists and to promote the guidelines’ dissemination through the use of new social media. (2) Methods: The survey was carried out during the COVID-19 lockdown in Italy (March–May 2020). The questionnaire was sent anonymously to a total of 600 dentists. The questionnaire consisted of two parts. Part A—demographic and professional data and Part B—management of traumatic avulsion. (3) Results: The response rate was 50.6% and the mean fraction of correct responses was 0.524. Issues related to the therapeutic management of avulsed teeth were shown to be not well understood by the respondents. Professionals with qualifications in dentistry and those who declared to know the guidelines responded better, while other demographic and professional factors were insignificant. (4) Conclusions: Italian dentists’ knowledge of the management of avulsion trauma should be improved. Educational programs and campaigns must be undertaken to improve their awareness and adherence to the Italian and international guidelines.

## 1. Introduction

Avulsion of permanent teeth is a common dental injury and it represents about 16% of dental injuries [1,2]. Maxillary central incisors are the teeth most frequently prone to avulsion [1]. Contact sports are the major cause of avulsion trauma with a higher prevalence among boys than girls [3]. Dental avulsion results in: (i) complete displacement of the tooth from its alveolus; (ii) injury to the soft tissues; (iii) fracture in the supporting bone [2].

In Italy, about one third and one fourth of all pre- and schoolchildren, respectively, have experienced a trauma to the permanent dentition, with a prevalence of 20.3% [4].

It is well established that the prognosis of avulsed teeth depends on appropriate management in the first moments after the trauma, according to the International Association of Dental Traumatology (IADT) guidelines, in order to ensure a standardization in the first aid procedures and a good long-term prognosis after replantation [1]. Schoolteachers and sports trainers as well as children’s parents are the first people involved in the initial decisions of treatment. Despite no agreement among lay people regarding the first place to be contacted after avulsion trauma, the majority would contact a nearby dentist rather than a general hospital directly [5]. In this scenario, the role played by a general practitioner dentist, oral and maxillofacial surgeon, pediatric dentist and orthodontist is of paramount importance in order to ensure a good prognosis and treatment outcomes of the replantation.

No recent data are available on the knowledge of management of avulsion trauma among Italian dentists.

The primary aim of this study was to investigate the knowledge of dental practitioners about the emergency management of avulsed teeth in Italy; the secondary aim was to promote the implementation of the guidelines’ dissemination through the use of new social media.

## 2. Materials and Methods

The target population was a group of general practitioner dentists (GPDs) in Italy. The study was approved by the Ethical Committee of Sapienza University of Rome (*n*.3493) and informed consent was obtained from all individuals. All the procedures were in accordance with the 1964 Helsinki Declaration and its later amendments or comparable ethical standards, for the protection of human subjects and animals in research. The survey was carried out during the COVID-19 lockdown in Italy, namely during the months of March, April and May 2020.

The questionnaire was developed using Google Forms and sent anonymously to a total of 600 dentists from the mailing list of the Italian scientific society “Accademia il Chirone”.

The questionnaire consisted of two parts. Part A—demographic and professional data, including training background (questions *n* = 11), and Part B—management of avulsed teeth (questions *n* = 13).

The queries related to Part 1, characteristics of the respondents (Q1: age; Q2: gender; Q3: professional experience; Q4: degree; Q5: specialty; Q6: PhD; Q7: academic affiliation; Q8: setting of work; Q9: formal training in avulsion trauma; Q10: personal experience in avulsion trauma; Q11: knowledge of the International Association of Dental Traumatology guidelines), are reported in Table 1.

The queries related to Part 2, management of avulsed teeth (Q1: How should an avulsed tooth be stored after the accident and before the reimplantation? Q2: If the avulsed tooth is dirty, how to clean it? Q3: How to treat the socket prior to replantation? Q4: What type of splint to stabilize the replanted tooth? Q5: Select splinting duration for extra-oral dry time < 60 min; Q6: Select splinting duration for extra-oral dry time > 60 min; Q7: If you want to do a root canal treatment, when should you start it? Q8: Which are the possible complications after tooth replantation? Q9: Patient arrives in your studio with an avulsed tooth. Would you replant it? Q10: In case of avulsion trauma, within how long should the patient seek specialist assistance? Q11: Would you do a root canal treatment of an avulsed and replanted tooth? Q12: Duration of follow up period; Q13: Is antibiotic therapy necessary after replantation?), are presented in Table 2.

The filled in questionnaires were coded by Google Forms, that generated an Excel database.

## 3. Results

### 3.1. Demographic Characteristics

A total of 304 dental practitioners participated to this study: 40.1% (*n*: 122) were female and 59.9% (*n*: 182) were male. The response rate was 50.6% of the contacted dental professionals answered the questionnaire.

The vast majority of the respondents were graduates in dentistry (82.2%) and 19.1% in medicine. The respondents with a medicine degree (*n*: 58) all presented a specialty: 57 in dentistry and related and one in pediatrics. The respondents with a dentistry degree (*n*: 250) had one or more specialties in 44.4% (*n*: 111) of the cases. Academic affiliation and PhD were declared by 23.7% (*n*: 71) and 10.5% (*n*: 32) of the respondents, respectively.

#### General Practitioners with a Degree in Medicine and Degree in Dentistry

In Italy in 1980, a special field of study in accordance with the European Union Directives was created. Persons with a medical and surgical degree obtained before this principle came into force were dependent on the exercise of a freelance profession and having a diploma of specialization in Dentistry. Then, in 1985, a distinction was made between the profession of a doctor-surgeon from that of a dentist, recognizing a separate university course for this profession.

Table 1 shows the respondents’ profile.

### 3.2. Response Rate

The knowledge of management of avulsed teeth was scored according to the answers to 13 questions. Eleven questions were scored 1 (correct answer) or 0 (wrong answer), while two questions had also fractional values between 0 and 1, due to the fact that they were multiple-choice questions.

Answers to the 13 questions on the management of teeth avulsion are shown in Table 2 [1].

The correct answers to each question are highlighted in italics.

Mean values of question scores are shown in Table 3 (in the case of 0–1 questions, mean = share of correct answers).

The first part of the questionnaire included questions (Q *n* 1, 2, 3) regarding the optimal storage media and the proper cleaning of the avulsed tooth and dental socket management prior to replantation.

The majority of the respondents indicated milk and saline (74.4%) and the patient’s mouth (52%) as the correct storage media for an avulsed tooth. Rinsing the avulsed tooth with saline was the correct answer for the question “How to clean a dirty avulsed tooth?”, given by 78.9% of the respondents. Gentle irrigation with saline was answered by 61.2% of the participants as the right pre-treatment of the socket prior to replantation.

The second part of the questionnaire included questions (Q *n* 4, 5, 6) regarding the proper splint to use to stabilize a replanted tooth. The incorrect response “rigid splint” was indicated by 66.8% of the participants, while 45.1% and 42.8% of the sample answered correctly on the splint duration for extra-oral dry time < 60 min and > 60 min, respectively.

The third part of the questionnaire was about the management of the tooth after replantation with questions regarding root canal treatment (RCT) complications (Q *n* 7, 8, 9, 11). The correct response ratio for questions regarding the root canal treatment (Q *n* 8 and 11) was low: 22.1% and 40.8%. Q7 was “If you want to do an RCT, when should you start it” and 63% of the participants answered that after replantation of an avulsed tooth they would monitor the tooth vitality and perform an RCT in patients who develop signs and symptoms. On the other hand, the correct ratio for possible complications was high, as 84.9% and 71.1% indicated root resorption and ankylosis, respectively.

The last part of the questionnaire included questions regarding follow-up duration (Q *n* 12) and the prescription of antibiotics (Q *n* 13). The correct response ratio was high, with 72.7% and 67.8%, respectively.

### 3.3. Statistical Analysis

Linear regression models were estimated for two variants of dependent variables: average score of 13 questions and weighted average score.

The initial models took into account:

- age (coded by middle of age intervals in corresponding question),
- gender (0—woman, 1—man),
- graduation (0—Medicine, 1—Dentistry),
- specialization (coded by three binary variables for dentistry, orthodontics, surgery present in the answer),
- years of experience (middle of interval, 25 if “>20” option was selected),
- PhD (0—No, 1—Yes),
- academic affiliation (0—No, 1—Yes),
- work (coded by two binary variables for mixed and private practice in comparison to public hospital),
- special training (0—No, 1—Yes),
- personal experience of teeth avulsion trauma treatment (0—No, 1—Yes),
- knowledge of guidelines (0—No, 1—Yes),

The best set of explanatory variables (final models) was selected on the basis of the adjusted R^2^.

#### Average Score

**Figure 1:**
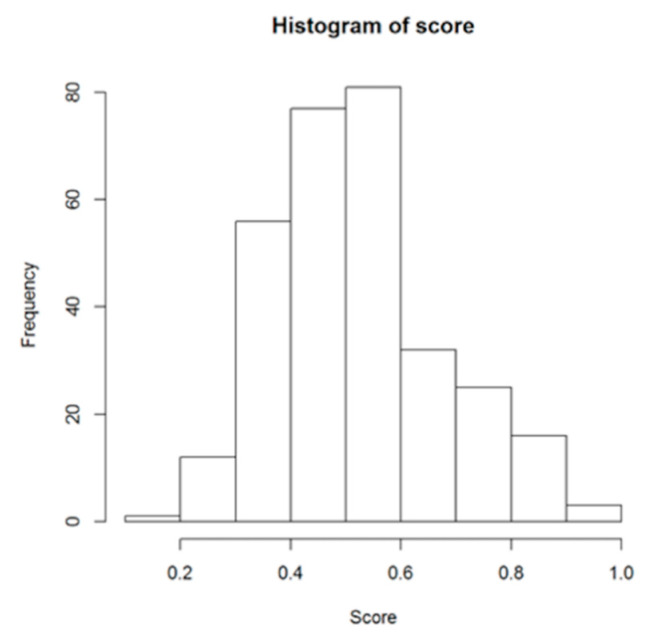
Histogram of average score distribution.

The total score of the dentists was calculated as a mean of 13 values and can thus take a value between 0 and 1. One outstanding value (score = 0) was excluded from consideration. A histogram of the scores is shown in Figure 1.

There are two significant characteristics—qualification in dentistry and knowledge of guidelines (Table 4).

The model is significant (F [4.298] = 5.947, *p* < 0.001), R^2^ = 0.074.

**Figure 2:**
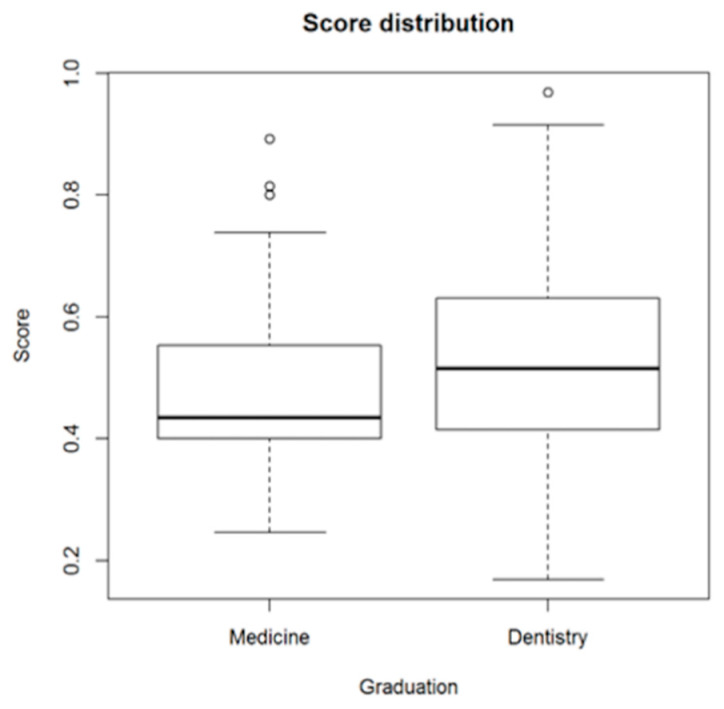
Distribution of average scores among dentists with medicine and dentistry degrees.

The model estimation result suggests that, ceteris paribus, the expected score of a dentist with a qualification in dentistry is 0.058 greater (almost one correct answer more) than in the case of a medicine qualification. There is a significant correlation between score and qualification, 0.144, *p* = 0.012 (Figure 2).

**Figure 3:**
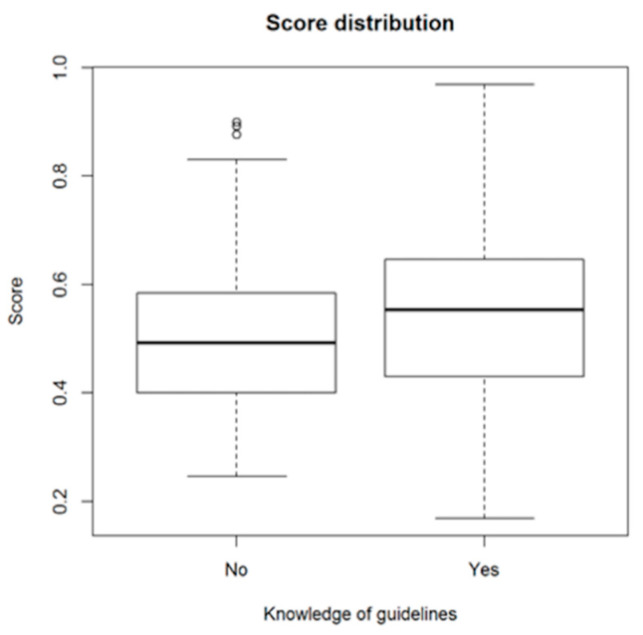
Distribution of average scores among doctors depending on knowledge of IADT guidelines declaration.

Similarly, knowledge of the guidelines adds 0.060 to the expected score. There is a significant correlation between the score and knowledge of IADT, 0.187, *p* = 0.001. Plots illustrate the score distribution in corresponding groups (Figure 3).

There are also two characteristics with insignificant coefficients in the final model—gender (correlation 0.092, insignificant, *p* = 0.111) and PhD (correlation 0.127, significant, *p* = 0.026). Models with some interactive variables were also considered (age or years of experience and qualification or specialization). The aim was to check if time taken to gain specialization has a significant influence on knowledge. These interactive variables did not improve the model.

## 4. Discussion

This survey, conducted during the COVID-19 lockdown in Italy, enrolled 304 respondents among dental practitioners. The response rate was 50.6% and the mean fraction of correct responses was 0.524. The sample was divided between dental professionals with medicine or dentistry degree. The aim of this survey was to assess the level of knowledge of the management of avulsed teeth among Italian dental professionals and to correlate it with years of experience and the dental curriculum.

The current survey showed that the storage medium, the suitable splint and the timing of root canal treatment of avulsed and replanted teeth were the critical steps of avulsion trauma management. In fact, the majority of respondents (74.7%) chose both milk and saline as the storage medium, but 52% chose saliva, which is not recommended by the IADT guidelines, as the risk of ingestion and inhalation exists (Q1).

After replantation, the tooth should be splinted. The results of this survey were not satisfactory, as 66.8% of the respondents would apply a rigid splint (Q4). The IADT guidelines suggest a flexible splint for a time of two weeks to decrease the risk of ankylosis [1]. Moreover, the IADT suggests to start the root canal treatment 7–10 days after replantation of an avulsed tooth with a closed apex and mature root, to prevent inflammatory tooth resorption and obtain a good prognosis over time. By contrast, the respondents answered that they would monitor the vitality of the replanted tooth (63%) and wait for signs and symptoms before deciding to perform an RCT (57.2%). In fact, in the present survey, only 22.1% of the enrolled respondents correctly answered the question on the timing of root canal treatment after avulsed tooth replantation (Q7). By contrast, the majority of respondents were aware of the possible complications of avulsed and replanted teeth, with 84.9% and 71.7% indicating, respectively, root resorption and tooth ankylosis. It is widely documented that the timing and nature of the endodontic treatment of a replanted tooth are crucial to avoid further tough complications [6,7,8,9,10].

The statistical analysis performed on average scores showed that:

- Professionals with dentistry degree perform better overall than those with medicine degree, as those that declare to know the IADT guidelines.
- Female dentists and those with academic affiliations had an overall slightly better knowledge.

Based on the findings of this study, there is a need to improve the knowledge of the management of teeth avulsion among Italian dental professionals, in accordance with the IADT guidelines [1]. Theoretical courses, specific training and continuing education programs both in the under- and post-graduate curriculum of dental schools, together with the evaluation of the acquired knowledge, are necessary to guarantee a high standard of care [11]. Moreover, dental practitioners in Italy should utilize the Italian guidelines for the prevention and management of dental trauma in children to obtain the appropriate information on evidence-based recommendations for the optimal prevention and treatment of avulsed teeth [12,13]. A multidisciplinary panel on the behalf of the Italian Ministry of Health and in collaboration with the WHO Collaborating Centre for Epidemiology and Community Dentistry of Milan developed this document [14]. In addition, as Barrett and Kenny underlined in their review, the prevention issue should be given greater emphasis through prophylactics and eliminating fall-prone areas, installing safety measures at homes, using protective appliances in sports, education and raising knowledge about and the availability of services to address dental trauma [15].

A comparison with data from similar surveys was carried out, and, despite differences in methodologies used to report on knowledge among dental professionals, the outcomes were interesting. Table 5 shows similar surveys conducted in different countries (USA, Brazil, Malaysia, Israel, Kuwait, UK, Germany, Belgium, Morocco, China) to assess the knowledge on dental trauma management among dental professionals.

The mean value of correct answers in the avulsion trauma management was always lower than the score reported for other dental trauma (crown and root fractures). The results of the current survey are in accordance to those represented in Table 5 [16,17,18,19,20,21,22,23,24,25,26,27,28].

A recent questionnaire study by Abdullah et al. conducted in Malaysia on a sample of 182 general dental practitioners (GDPs) defined the level of knowledge as adequate with a mean correct response rate of 72.7% [16]. These results, although considered to be good, were nevertheless identified as to be improved.

In fact, a score of 72.7% can be defined as an adequate level of knowledge, however, it does not ensure an adequate level of competence in the entire teeth avulsion protocol in order to achieve a good long-term prognosis. Indeed, a level of knowledge which is not complete in all aspects cannot be considered sufficient to guarantee optimal results.

The reported scores by Abdullah et al. were the highest [16] compared to the scores reported by Zadik [17] and Hartmann [18], with an average correct response rate of 61.5% and 60.2%, respectively.

As reported by Krastl, the treatment of dental trauma is a rare event in private practice and it is normal that GDPs are not confident in its management. Krastl conducted a survey in Germany and the vast majority of the respondents judged the frequency of dental trauma as very rare and they were unable to assess their own level of knowledge [19].

De Franca reported the results of a survey conducted in Brazil, where the correct response rate on the emergency management of avulsed teeth ranged between 16.1% and 36.6%, and the author concluded that the great majority of dental professionals would not intervene according to the literature guidelines [20].

The study by Cohenca et al. conducted in 2006 with 167 GDPs attending continuing education courses at the School of Dentistry, University of Southern California, documented a correct response rate on the trauma management of teeth avulsion of 54.6%. The authors pointed out that, although results may be similar to previous studies, extrapolation of the outcomes to other oral health providers in the USA or in other countries should be done with extreme caution [21].

Standardization in methodology is needed and recently the updated IADT guidelines underlined the development of a core outcome set (COS) for traumatic dental injuries in children and adults [1]. Further dissemination will help clinicians and researchers to implement it.

The results of the present survey are in accordance with Kostopoulou et al. who showed in a sample size of 693 respondents that those who were younger and more recently graduated performed better than the others [22]. Cohenca et al. also showed a similar pattern, pointing out that those who were more experienced and those who had attended continuing education courses within the last 3 years responded more correctly [21].

The COVID-19 pandemic has called upon us to do things differently and education and teaching are going to change, as direct contact between those who learn and those who teach is not allowed on a regular basis. The development of online courses and webinars, regularly scheduled during the dental undergraduate and postgraduate programs, and continuing education, should be promoted. In fact, Zhao et al. showed a great difference in dental postgraduate education between urban and suburban dentists: 31.3% of urban dentists presented a master’s or a PhD degree compared to 6.8% of the suburban dentists. Zhao et al. in accordance with the position of this paper, underlined that no difference was present between the two categories concerning continuing education programs [23].

Webinars do not create discrimination in access to education, and continuing education is of paramount importance. Moreover, webinars are interactive, easy to use, they reach a large audience, are repeatable and are inexpensive, if compared with traditional courses [23]. The webinar, in this historical moment, seems to represent the most suitable tool for the widespread dissemination of the IADT guidelines.

Therefore, according to the aims of this study and, consequently, the results of the survey on the knowledge of the emergency management of the avulsion of permanent teeth among Italian dental practitioners, a webinar on dental trauma in pediatric population was developed by the Italian Society of Odontostomatology and Maxillofacial Surgery—Società Italiana di Odontostomatologia e Chirurgia Maxillo-Facciale (SIOCMF) and the Italian Society of Pediatric Emergency Medicine—Società Italiana di Medicina Emergenza Urgenza Pediatrica (SIMEUP) and it is fully available at: https://www.siocmf.it/corsi-e-congressi.html and at: https://www.simeup.it/?page_id=16531.

## 5. Conclusions

Based on the findings of this survey, Italian dentists’ knowledge of the management of avulsed teeth should be improved. Educational programs and campaigns must be undertaken to improve their awareness and adherence to the Italian and international guidelines.

## Figures and Tables

**Table 1:** Characteristics of the respondents.

Questionnaire Part A: Respondents’ Characteristics		
Queries	Answers	*n*
Q1: Age	20–39 (59.5%)	181
	40–49 (13.2%)	40
	50–69 (27.3%)	83
Q2: Professional experience	0–5 years (27%)	82
	6–10 years (18.1%)	55
	11–15 years (19.7%)	60
	16–20 years (5.3%)	16
	>20 years (29.9%)	91
Q3: Sex	Women (40.1%)	122
	Men (59.9%)	182
Q4: Degree	Medicine (19.1%)	58
	Dentistry (82.2%)	250
Q5: Specialty	Pediatrics	1
	Dentistry	57
	Orthodontics	76
	Pediatric Dentistry	13
	Oral Surgery	44
	Maxillofacial Surgery	2
Q6: PhD	Yes (10.5%)	32
	No (89.5%)	272
Q7: Academic affiliation	Yes (23.7%)	71
	No (76.3%)	232
Q8: Setting of work	Public hospital (4.9%)	15
	Private practice (81.6%)	248
	Mixed (PH + PP) (16.1%)	49
Q9: Formal training in avulsion trauma	During degree (47.7%)	145
	After degree (28.9%)	88
	Self-education (23.4%)	71
Q10: Personal experience in avulsion trauma	Yes (48.7%)	148
	No (51.3%)	156
Q11: Knowledge of the guidelines of International Association of Dental Traumatology (IADT)	Yes (43.4%)	132
	No (56.6%)	172

**Table 2:** The answers given by the respondents. The correct answers are marked in italics.

Questionnaire Part B: Management ofAvulsed Teeth		
Queries	Answers	*n*
Q1: How should an avulsed tooth be stored after the accident and before the reimplantation?	No storage necessary (0.3%)	1
	Tap water (4.6%)	14
	Cold water (0.3%)	1
	Water and salt (4.6%)	14
	Ice (2.3%)	7
	Coca-cola (0.3%)	1
	*Milk* (74.7%)	227
	Saline solution for contact lenses (16.8%)	51
	*Saline* (74.7%)	227
	Patient’s mouth (52%)	158
	Keep the tooth in gauze (2%)	6
	Keep the tooth in plastic film (0.3%)	1
Q2: If the avulsed tooth is dirty, how to clean it?	Wash tooth in water (6.3%)	19
	*Rinse the tooth in saline* (78.9%)	240
	Scrub the tooth gently with a toothbrush (2%)	6
	Keep the tooth for 10 min in a NaFl solution (12.8%)	39
Q3: How to treat the socket prior to replantation?	*Gentle irrigation and aspiration with saline* (61.2%)	186
	Removal of coagulum with courettes (12.5%)	38
	No treatment (26.3%)	80
Q4: What type of splint to stabilize the replanted tooth?	Rigid splint (66.8%)	203
	*Flexible splint* (31.6%)	96
	No splinting (1.6%)	5
Q5: Select splinting duration for extra-oral dry time < 60 min	*10–14 days* (45.1%)	137
	28–30 days (38.2%)	116
	60 days (14.1%)	43
	No splinting (2.6%)	8
Q6: Select splinting duration for extra-oral dry time > 60 min	*10–14 days* (13.8%)	42
	28–30 days (42.8%)	130
	60 days (39.8%)	121
	No splinting (3.6%)	11
Q7: If you want to do a root canal treatment, when should you start it?	Immediately after replanting the tooth (10.6%)	32
	*7–10 days after replantation* (22.1%)	67
	1 month after replantation (7.9%)	24
	Yes, I will monitor tooth vitality and I will perform a root canal treatment (RCT) after patient shows signs and symptoms (63%)	180
Q8: Which are the possible complications after tooth replantation?	Coronal resorption (2.6%)	8
	*Radicular resorption* (84.9%)	258
	Granuloma (27.6%)	84
	*Pulp necrosis* (69.1%)	210
	*Ankylosis* (71.1%)	216
Q9: Patient arrives in your studio with an avulsed tooth. Would you replant it?	Yes, in all cases (22.4%)	68
	*Yes, only for permanent teeth* (72.7%)	221
	No (1.6%)	5
	I refer teeth avulsion cases to hospital/specialist (3.3%)	10
Q10: In case of avulsion trauma, within how long should the patient seek specialist assistance?	*Immediately* (81.3%)	247
	0–30 min (8.2%)	25
	30–60 min (4.6%)	14
	In the first 6 h (3.3%)	10
	In the first 24 h (1.6%)	5
	No need for specialist care (1%)	3
Q11: Would you do a root canal treatment of an avulsed and replanted tooth?	Yes, if the tooth is immature with an open apex (2%)	6
	*Yes, if the tooth has a fully formed and closed apex* (40.8%)	124
	Yes, only after patient develops signs and symptoms (57.2%)	174
Q12: Duration of follow up period	*Clinical and radiographic examinations at 1, 3, 6 and 12 months* (72.7%)	221
	Clinical and radiographic examinations at 1, 6, 12 and 24 months (19.4%)	59
	Clinical and radiographic examinations every 6 months up to 36 months (6.9%)	21
	No need for follow-up (1%)	3
Q13: Is antibiotic therapy necessary after replantation?	*Yes* (67.8%)	206
	Yes, only topical antibiotics (5.3%)	16
	No (27%)	82

**Table 3:** Mean fraction of correct responses to each question.

Question	Mean
1	0.412
2	0.795
3	0.614
4	0.320
5	0.452
6	0.429
7	0.221
8	0.209
9	0.729
10	0.815
11	0.409
12	0.733
13	0.680
Average	0.524

**Table 4:** Model estimation for average score.

	Estimate	Std. Error	*t* Value	*p* = Pr(>|t|)	
(Intercept)	0.4608	0.027	17.210	0.000	***
Gender	−0.0225	0.018	−1.235	0.218	
Qualification in dentistry	0.0584	0.023	2.551	0.011	*
PhD	0.0402	0.029	1.367	0.173	
Knowledge of guidelines	0.0601	0.018	3.319	0.001	**

*—*p* < 0.05, **—*p* < 0.01, ***—*p* < 0.0001.

**Table 5:** Summary of studies included in the Discussion.

Study	Author	Journal	Year	Survey Area	Survey Setting	Sample Size	Reported Knowledge	
							Dental Trauma	Avulsion Trauma
A study into dentists’ knowledge of the treatment of traumatic injuries to young permanent incisors	M. N. Kostopoulou & M. S. Duggal [22]	Int. J. Paediatr. Dent.	2005	West/North Yorkshire and Humber-side, UK	General dental practitioners (GDPs) and community dental officers (CDOs)	693	NR CDOs significantly more knowledgeable than younger and more recently graduated dentists	Storage media, splinting GDPs: 59%, 26% CDOs: 70%, 53%
Knowledge of Brazilian general dentists and endodontists about the emergency management of dento-alveolar trauma	L.W. Hu [24]	Dent Traumatol	2006	Brazil	GDPs endodontists	98 44	Dental trauma 64% (GDPs); 77% (endo) this survey showed a poor knowledge of dental trauma management among the surveyed dentists and highlights the need to develop strategies to improve the knowledge base in this area of dentistry for the benefit of the dental trauma patient	59% semirigid splint
Knowledge of oral health professionals of treatment of avulsed teeth	N. Cohenca [21]	Dent Traumatol	2006	The School of Dentistry, University of Southern California	General dentists attending Continuing Education courses at	167	NR Experienced dentists and those with education in dental trauma within the last 3 years	54.6%
Knowledge of general practitioners dentists about the emergency management of dental avulsion in Curitiba, Brazil	V. P. Westphalen [25]	Dent Traumatol	2007	Curitiba, Brazil	General practitioner dentists	250	NR Reporting adequate level of knowledge	51.14%
Knowledge of emergency management of avulsed teeth among young physicians and dentists	M. Abu-Dawoud [26]	Dent Traumatol	2007	Kuwait	Newly graduated physicians Dentists	30 30	Physicians could not provide first aid care in case of dental trauma, medium level of knowledge reported among dentists	NR
Brazilian dentists’ knowledge regarding immediate treatment of traumatic dental injuries	R. I. de Franca [20]	Dent Traumatol	2007	Tubarao, Brazil	Dental surgeons	93	Coronal fractures 75.3% Luxations 73.1% Those with <10 years of experience performed better	Avulsions 16.1–36.6%
German general dentists’ knowledge of dental trauma	G. Krastl [19]	Dent Traumatol	2009	Germany	General dentists	168	Dental trauma 29.7% Generally poor knowledge among general dentists in Germany on dental traumatology. General practitioners were unable to accurately assess their own level of knowledge	Avulsion 42.05%
Dental practitioners’ knowledge and implementation of the 2007 International Association of Dental Traumatology guidelines for management of dental trauma	Y. Zadik [17]	Dent Traumatol	2009	Israel	Medical Corps of the Israel Defense	54	Overall 71.7% Root fracture 61.1% High level of knowledge regarding the 2007 guidelines among Israeli military dentists. Knowledge regarding several issues (e.g., medication, splinting) should be reinforced	Avulsion 60.2%
Knowledge of emergency management of avulsed teeth: A survey of dentists in Beijing, China	Y. Zhao & Y. Gong [23]	Dent Traumatol	2010	Beijing, China	Urban and suburban dentists	175 99	Uneven pattern of knowledge between urban and suburban dentists	Storage medium 15.8%; type of splint 45.1%; splinting period 10.2%; intracanal medication 45%
Educational background of Flemish dental practitioners and their perceptions of their management of dental trauma	R. G. Cauwels [27]	Dent Traumatol	2014	Belgium	Dental practitioners	336	Answers given for the treatment of injured incisors with open apices and a large pulp exposure, 23% knowledge of Flemish dental practitioners regarding emergency treatment for CCF is insufficient	NR
Knowledge of managing avulsed tooth among general dental practitioners in Malaysia	D. Abdullah [16]	Singapore Dent J	2016	Malaysia	General dental practitioners	182	Need to be improved	Avulsion 72.7%
Knowledge of Moroccan dentists about the management of dental hard tissue trauma	S. Dhaimy [28]	OHDM	2017	Morocco	General dental practitioners	205	Dental trauma 59%	NR
Dentists′ knowledge of dental trauma based on the International Association of Dental Traumatology guidelines: A survey in South Brazil	R. C. Hartmann [18]	Dent Traumatol	2018	Brazil	General dental practitioners	1414	Crown fractures/enamel/dentine: higher % correct answers	Avulsion 61.5%

## Data Availability

The data presented in this study are available on request from the corresponding author.
